# Estimated prevalence and viral transmissibility in subjects with asymptomatic SARS-CoV-2 infections in Wuhan, China

**DOI:** 10.1093/pcmedi/pbaa032

**Published:** 2020-09-04

**Authors:** Kang Zhang, Weiwei Tong, Xinghuan Wang, Johnson Yiu-Nam Lau

**Affiliations:** Center for BioMedicine and Innovations, Faculty of Medicine, Macau University of Science and Technology, Macao, China; Center for BioMedicine and Innovations, Faculty of Medicine, Macau University of Science and Technology, Macao, China; Zhongnan Hospital of Wuhan University, Wuhan 430071, China; Department of Applied Biology and Chemical Technologies, Hong Kong Polytechnic University, Hung Hom, Hong Kong SAR, China

**Keywords:** asymptomatic carriers, infection, COVID-19

## Abstract

The role of subjects with asymptomatic SARS-CoV-2 infection in the current pandemic is not well-defined. Based on two different approaches to estimate the culminative attack rate (seroprevalence of antibodies against SARS-CoV-2, and a four compartment mathematical model) and the reported number of patients with COVID-19, the ratio of asymptomatic versus symptomatic SARS-CoV-2 infection was estimated to be 7 (95% CI: 2.8–12.4) in Wuhan, Hubei, China, the first epicenter of this pandemic, which has settled with no new cases. Together with detailed recording of the contact sources in a cohort of patients, and applying the estimations to an established mathematical model, the viral transmissibility of the subjects with asymptomatic SARS-CoV-2 infection is around 10% of that of the symptomatic patients (95% CI: 7.6%–12.3%). Public health measures/policies should address this important pool of infectious source in combat against this viral pandemic.

The role of subjects with asymptomatic SARS-CoV-2 infection in the current pandemic is not yet clear. Early in the pandemic (reported between 10 February and 24 April 2020), the ratio of asymptomatic to symptomatic subjects with SARS-CoV-2 infection was estimated to be 0.22–1.27 times based on viral nucleic acid RT-PCR assay (Fig. 1). Subsequent studies based on serology for antibodies to SARS-CoV-2, an indicator of immune response related to a past exposure (and reported from 23 April to 3 June 2020) gave a much higher estimation of 8.46–10.24 times (Fig. 1). The timing of the study, the study population, and the performance of assays could all impact assessment of the prevalence and, as a result, the impact of asymptomatic SARS-CoV-2 infection in its contribution to this pandemic.

**Figure 1.**
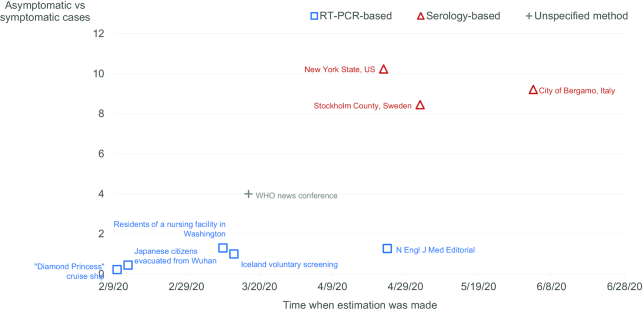
Various estimations of the ratio between asymptomatic and symptomatic SARS-CoV-2 infections and the time when these estimations were made. Squares represent estimations based on viral RT-PCR test results. Triangles represent estimations based on serology test results in various cities or regions. Cross represents estimation based on unspecified data source.

Wuhan City in Hubei Province, China was the first epicenter of this pandemic. The situation in the city has settled, with no new local cases reported for more than two months, and, therefore, there is an opportunity to look back and determine the prevalence and role of asymptomatic SARS-CoV-2 infection in this pandemic. Timely understanding of the role of asymptomatic SARS-CoV-2 infection is critical as such scientific information could be used by communities that are still in the midst of the pandemic to finetune their public health measures/policies. Therefore, we attempted to estimate the prevalence of asymptomatic SARS-CoV-2 infection (using two different approaches), the viral transmissibility, the contribution of this pool of infected subjects to the pandemic, and the implications of this information on public healthcare measures/policies.

First, with reference to prevalence, we employed two different approaches to estimate the number of subjects with asymptomatic SARS-CoV-2 infection in Wuhan. We have previously shown a cumulative prevalence of COVID-19 of ∼3.2%–3.8% in Wuhan, with estimates based on a survey of serum IgM and IgG levels collected between 9 March 2020 and 10 April 2020 from 17 368 residents in the city.^1^ Based on another survey of 16 101 community subjects who underwent health check-ups between 15 March 2020 and 7 May 2020 in another hospital in Wuhan, a total of 709 (4.4%) subjects was found to be seropositive for either IgG (*n* = 539) or IgM (*n* = 64), or both IgG/IgM (*n* = 106) against SARS-CoV-2. Therefore, the seroprevalance for SARS-CoV-2 antibodies, or exposure to this virus, is confirmed to be around 4% in Wuhan. Using a different approach, we employed our previously published mathematical model based on a four-compartment modified SQIR (susceptible-quarantined-infected-removed) approach, with the infected compartment subdivided into different statuses to describe latent (infected but not contagious), asymptomatic (not showing symptoms or never showing symptoms), and symptomatic individuals^2^ (Fig. 2). Based on the Wuhan screening data and our model (Figs. 2 and 3), we estimate that during the entire course of the outbreak in Wuhan, there was a total of 398 346 infected patients (95% CI: 196 002–684 777), or 3.59% (95% CI: 1.77%–6.17%) of the city's population. Both seroprevalence and mathematical modeling approaches provided consistent prevalence figures for SARS-CoV-2 infection of 3.2%–4.4%, or around 400 000 subjects in total in Wuhan with a population of 11 million. As there were 51 081 symptomatic subjects as reported by Chinese CDC in Wuhan, the balance of 349 000 subjects will be those with asymptomatic SARS-CoV-2 infection. Therefore, the ratio of subjects with asymptomatic SARS-CoV-2 versus symptomatic COVID-19 patients was around 7 (with our mathematical model having 95% CI: 2.8–12.4).

**Figure 2.**
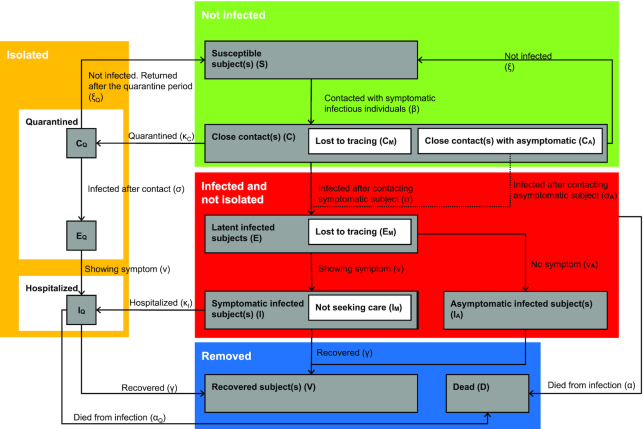
Revised flow diagram of the four-compartment mathematical model of disease transmission, which incorporates the viral transmissibility and the impact of quarantine and social distancing (revised from our previous model cited in reference ^2^). The population is divided into the following states: susceptible subject(s) (S), had close contact(s) (C), those that were exposed to the infectious subjects/pathogen but not necessarily infected (ξ) and (ξQ), latent infected subject(s) (E, infected but not infectious), symptomatic infectious subject(s) (I), asymptomatic infectious subject(s) (I_A_), recovered (V), and dead (D). C_M_ is the portion of the contact cases that is missed by contact tracing. C_A_ is the portion of the cases contacted with asymptomatic infectious subjects (thus remaining unknown). Both C_M_ and C_A_ will not be quarantined. Individuals in states C and C_A_ will progress to latent group E. When latent subjects become infectious, the symptomatic subjects are moved to the infectious status I, and asymptomatic to I_A_. C_Q_, E_Q_, and I_Q_ denote subjects in the quarantine facilities or isolation wards who are quarantined but not necessarily infectious, latent infected, and infectious, respectively. As all subjects under quarantine were regularly tested for SARS-CoV-2, all infected subjects were hospitalized regardless of being symptomatic or asymptomatic. It was assumed that when the infected subjects had recovered, they would acquire immunity that did not wane during the timeframe of the analysis (i.e. of this season).

**Figure 3.**
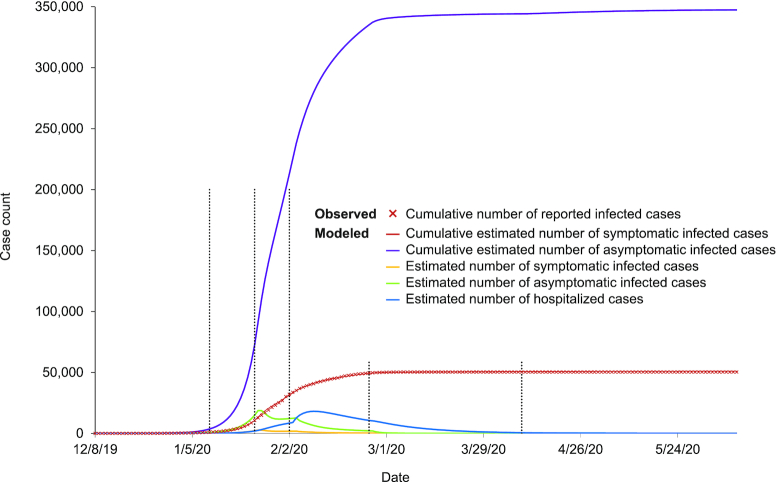
Observed and estimated case epidemic trajectories in Wuhan, Hubei, China. A cross symbol represents the cumulative numbers of cases observed. Curves represent the model fitted to the observed data using Maximum Likelihood Estimations (red curve represents the cumulative model-estimated numbers of symptomatic cases, purple curve represents the cumulative model-estimated numbers of asymptomatic cases). Six distinct periods were defined: (a) prior to January 10, 2020 before major public health interventions; (b) between January 10 and January 23, 2020, when moderate public health intervention [which would lower per͏͏͏͏ capita contact rates (β)] including travel bans were in place; (c) between January 23 and February 2, 2020, when there was travel ban and cancellation of social gatherings [which would further lower per͏͏͏͏ capita contact rates (β)] and compulsory facemask use [which would lower the infection rate upon contact (σ)]; (d) between February 2 and February 25, 2020, when quarantine was in place with limited capacity; (e) between February 25 and April 9, 2020, when quarantine was in place with full scale contract tracing and sufficient capacity; and (f) after April 9, 2020, when travel ban was lifted.

Second, we evaluated in detail the contact sources of COVID-19 vs non-COVID-19 patients in Wuhan from Zhongnan Hospital of Wuhan University. Out of 212 patients with COVID-19 admitted to the hospital after the city lockdown, 43.8% had confirmed exposure history (defined as having visited Huanan Seafood Wholesale Market, contact with patients with COVID-19, or residing in the same household as a patient with COVID-19 diagnosed before or after). Therefore, 56.2% of the patients with COVID-19 who had no exposure history might have acquired their viral infection through contact with subjects who had an asymptomatic SARS-CoV-2 infection. In contrast, of 571 non-COVID-19 patients, only 17.1% had similar exposure history. More specifically, non-COVID-19 patients represented patients who were admitted at the same time for other medical problems and confirmed not to have COVID-19 and 17.1% had exposure history to COVID-19 patients/subjects/environment in this epidemic area based on history taking but confirmed that they did not acquire COVID-19.

Third, in a revised mathematical model,^2^ we assumed that the viral transmissibility of asymptomatic infectious subjects (σ_A_) was discounted at a fixed ratio relative to that of symptomatic infected subjects (σ), and both were affected by general facemask use policy. Using the estimated number of subjects with asymptomatic SARS-CoV-2 infection and the actual number of infections as additional input (Fig. 2), we were able to estimate the viral transmissibility (or transmission efficiency) of the subjects with asymptomatic SARS-CoV-2 infection to be around 10% (95% CI: 7.6–12.3%) of that of the symptomatic patients with COVID-19. This is consistent with the hypothesis that subjects who can mount an earlier immune response can control the viral infection better and therefore with less viral load for viral transmission to others. In contrast, subjects without a good early immune response will allow the viral infection to establish a large viral load, which will render the patients more infectious and cause more cellular/tissue damage, consequently providing a much severe response later during the clinical course. If our hypothesis is correct, then the reported greater immune response in patients with COVID-19 is more a consequence of an earlier weaker response and therefore, a larger viral load and subsequently a higher immune response and tissue damage.^3^ The claim of a weaker immune response in asymptomatic individuals in a recent paper, is therefore, the cart rather than the horse.^3^

With an estimated ratio of 7x more asymptomatic infection, together with a transmission factor of 10% viral transmissibility compared to symptomatic cases, this suggests that the contribution of subjects with asymptomatic SARS-CoV-2 infection represents a significant source for new infections. This is consistent with a report that asymptomatic subjects have a lower viral load when first identified but, importantly, the duration of viral shedding is similar to that of patients with COVID-19.^4^ Public measures/policies to limit their infection spread once identified will be critical to contain these infection sources. We have previously estimated the impacts of individual public health interventions separately based on the epidemic curves of multiple countries.^2^ Both general facemask use (through lowering σ_A_) and social distancing/lockdown (through lowering β) are effective in mitigating the spread of SARS-CoV-2 from infectious individuals who are asymptomatic, the “dark mass” in the perpetuation of this pandemic.

In addition, if the ratio of 7 asymptomatic to 1 symptomatic SARS-CoV-2 infections is confirmed and applicable to other countries, as of 19 July 2020 (date of submission of this manuscript), the estimated cumulative number of SARS-CoV-2 infections in the US will be close to 30 million, in Brazil around 16 million, and worldwide around 100 million.

To corroborate with another survey in Wuhan,^5–7^ our mathematical model estimated that there were still around 897 infected subjects two weeks after the last reported case in Wuhan on 18 May 2020. Indeed, a government survey in May 2020 on 9.9 million Wuhan residents identified around 300 RT-PCR positive cases.^8^ Interestingly, all 1174 close contacts of these RNA positive cases were found to be negative for SARS-CoV-2. Whether these positive cases were infectious and the infection limited by good public polices and general facemask use, which reduce the viral transmissibility factor (based on our model), or whether these subjects only had viral RNA residues or defective interfering particles, remains unknown.
